# Induced Pluripotent Stem Cells (iPSCs)—Roles in Regenerative Therapies, Disease Modelling and Drug Screening

**DOI:** 10.3390/cells10092319

**Published:** 2021-09-05

**Authors:** Mourad A. M. Aboul-Soud, Alhusain J. Alzahrani, Amer Mahmoud

**Affiliations:** 1 Chair of Medical and Molecular Genetics Research, Department of Clinical Laboratory Sciences, College of Applied Medical Sciences, King Saud University, Riyadh 11433, Saudi Arabia; 2 Department of Clinical Sciences, College of Applied Medical Sciences, University of Hafr Al Batin, Hafr Al Batin 39524, Saudi Arabia; ajalzahrani@uhb.edu.sa; 3 Stem Cell Unit, Department of Anatomy, College of Medicine, King Saud University, Riyadh 11461, Saudi Arabia; ammahmood@ksu.edu.sa

**Keywords:** drug screening, disease, embryo, induced pluripotent stem cells, modelling

## Abstract

The discovery of induced pluripotent stem cells (iPSCs) has made an invaluable contribution to the field of regenerative medicine, paving way for identifying the true potential of human embryonic stem cells (ESCs). Since the controversy around ethicality of ESCs continue to be debated, iPSCs have been used to circumvent the process around destruction of the human embryo. The use of iPSCs have transformed biological research, wherein increasing number of studies are documenting nuclear reprogramming strategies to make them beneficial models for drug screening as well as disease modelling. The flexibility around the use of iPSCs include compatibility to non-invasive harvesting, and ability to source from patients with rare diseases. iPSCs have been widely used in cardiac disease modelling, studying inherited arrhythmias, neural disorders including Alzheimer’s disease, liver disease, and spinal cord injury. Extensive research around identifying factors that are involved in maintaining the identity of ESCs during induction of pluripotency in somatic cells is undertaken. The focus of the current review is to detail all the clinical translation research around iPSCs and the strength of its ever-growing potential in the clinical space.

## 1. Introduction

**Figure 1:**
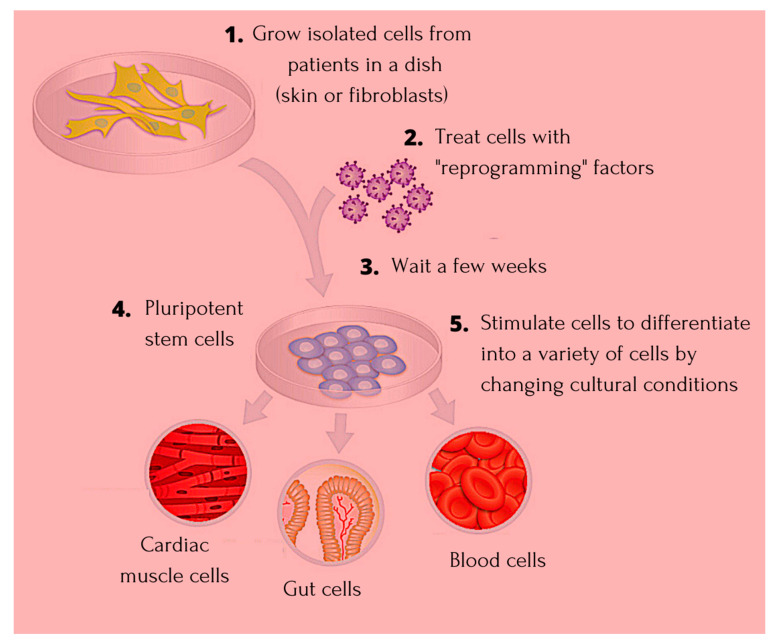
Showing the process of progression and generating iPSC cells. Detailed description of creating iPSCs with reprogramming factors and differentiating them into a variety of cell types.

The science around terminal inactivation and deletion of genetic codes of heredity in somatic cells was postulated by the Weismann barrier theory [1]. The somatic cell nuclear transfer (SCNT) demonstration asserted the fact that the genetic code in somatic cells is not discarded, and that reactivation of the same is a possibility through careful manipulations [2]. Developmental biology entered a new dimension of achievement when the discovery of embryonic stem cells (ESCs) and their pluripotency was exhibited, and further research identified that on fusion of somatic cells like fibroblasts, and T-lymphocytes with ESCs, reprogramming of the former through expression of genes associated with pluripotency becomes a possibility [3,4]. The findings around SCNT and ESC fusion identified the possibility of reversion in somatic cells indicating the presence of reprogramming factors that bear the potential to act as epigenetic memory erasing factors [5]. The earliest study around generation of pluripotent stem cells from fibroblasts was linked to introduction of four crucial transcription factors including octamer binding transcription factor 3/4 (Oct3/4), sex determining region Y—box 2 (SRY-Sox2), Krüppel-like factor 4 (Klf4), and cellular-Myelocytomatosis (c-Myc) (OSKM) [6]. The allogenic trait of ESCs, risk of immune rejection in the recipient along with need for lifetime immunosuppression, and the ethicality around using the same, makes human induced pluripotent stem cells (iPSCs) an established candidate for regenerative therapies as they were found to not impact the host immune system [7]. The introduction of the iPSCs technology happened in the year 2006, and since then multiple observational studies have recounted its impact on cardiac diseases, ophthalmic conditions, as well as neurological disorders [8,9,10]. Figure 1 highlights the process of generating iPS cells.

The nuclear reprogramming strategies, without compromising on safety and quality for therapeutic applications, include the integrative or nonintegrative transfer systems using viral or nonviral vectors. The first iPSCs were generated by integrating viral vectors, more popularly the retrovirus wherein the resultant iPSCs exhibited failure in complete expression of endogenous genes of pluripotency [11]. The more efficient viral vector has been documented to be the lentiviral vector (LV), which has recorded a reprogramming efficiency of between 0.1–1% [12,13,14]. To ensure increased safety for therapeutics, nonviral integrative systems have also been worked upon involving use of two plasmids; once encoding for c-Myc, and the other for the four reprogramming factors [15]. However, this system was also shown to have risk of integration, and low reprogramming efficiency. In case of nonintegrative nonviral systems for reprogramming, delivery of pluripotency marker genes has been done using self-replicating vectors, and cytoplasmic RNA. Though easy to work with, the reprogramming efficiency has been found to be lower than LV [16]. Today, research has identified possibility of successful reprogramming using microRNAs (miRNAs) which exhibit improved efficiency, wherein use of *c-Myc* has been replaced with *miR-291-3p*, miR-294, and *miR-295* to generate homogenous colonies of human iPSCs [17]. The reprogramming methods have been highlighted in Table 1.

**Figure 2:**
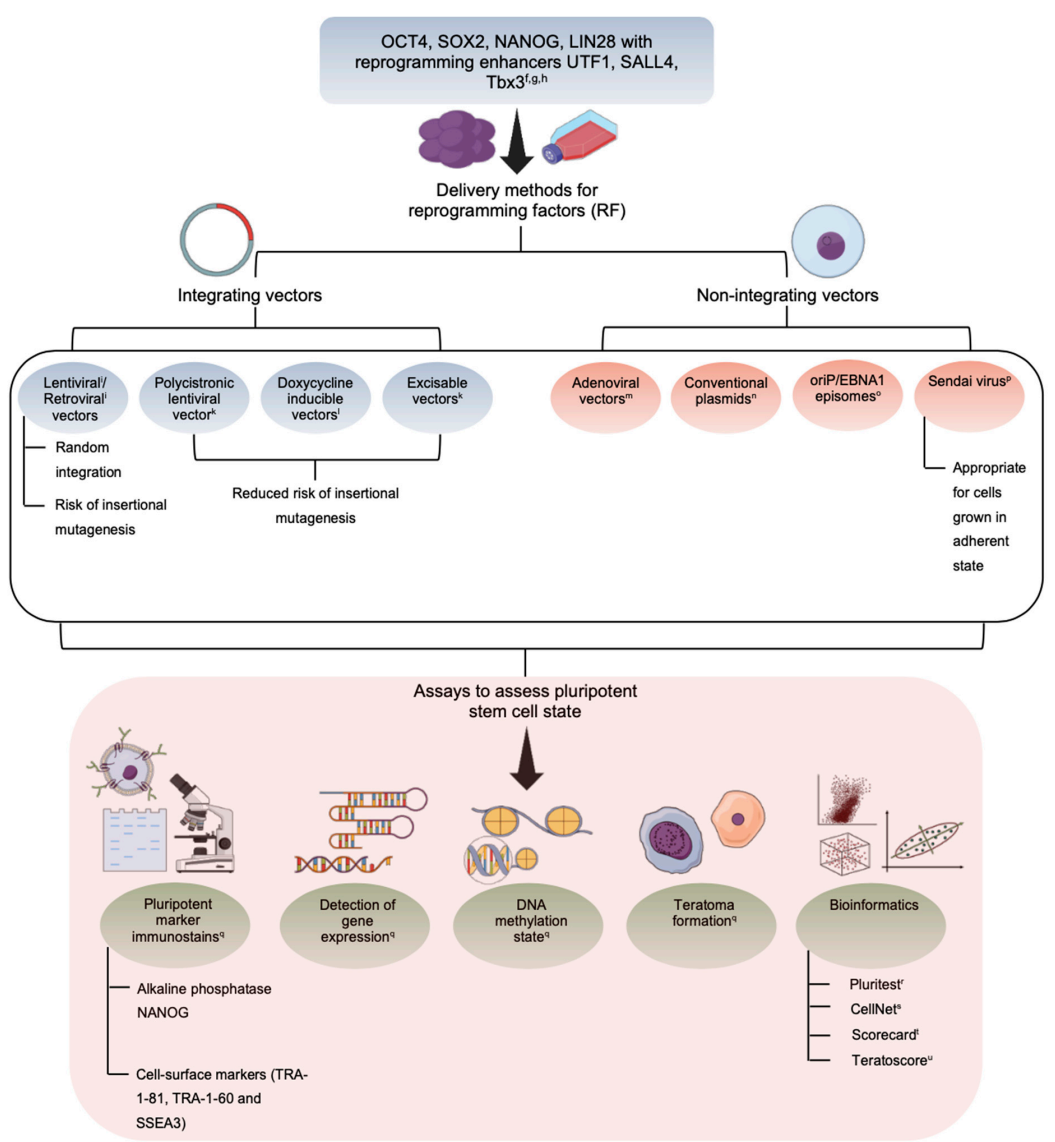
Schematic representation on derivation and assay for human iPSCs. Detailed schematic representation of derivation of iPSC with the various assays to evaluate the developmental efficiency.

There are many assays, including molecular and functional, to evaluate the developmental efficiency of iPSCs. These include alkaline phosphatase staining of pluripotency markers, DNA demethylation, retroviral silencing, and factor independence involving assessment of self-renewal in the absence of dox-inducible *trans* genes. The functional assays include teratoma formation, chimera development, tetraploid complementation, germline transmission, and in vitro differentiation [14]. Considering the low reprogramming efficiency in iPSCs, many studies have identified blocks in lineage conversion. Reprogramming pathway studies in fibroblasts have identified the repel factor to be involved in mesenchymal-to-epithelial transition (MET) and BMP receptor signaling [27,28]. Further studies on the refractory fibroblasts indicate negative iPSC generation in spite of prolonged culturing and presence of homogeneous factor expression indicating loss of somatic program, and activation of endogenous pluripotency genes to be the main roadblocks in formation of iPSCs [14]. The other limiting factor has been linked to expression levels of Nanog locus which are activated late in the reprogramming process and thus limit efficiency of conversion [29]. Gene silencing by DNA methylation, involving the pluripotency genes nanog and *Oct4* which causes blockage in binding of transcription factors, has also been linked to causing interference in reprogramming [30]. Though the four most popular reprogramming factors have been Oct4, Sox2, Klf4, and c-Myc, human iPSCs have also been derived using expression of Oct4, Sox2, Nanog, and Lin28, indicating that pluripotent ground state becomes achievable through activation of different transcription factors [21]. The detailed derivation of iPSC along with the assay has been highlighted in Figure 2.

The therapeutic potential of iPSC towards personalized cell therapy and disease modelling, has extended the functionality beyond laboratory tables as a research tool in murine and human models. Animal studies have identified promising potential of iPSC around treatment of genetic disorders, including sickle cell anemia; disease modelling of complex degenerative conditions like diabetes, Alzheimer’s disease, and the feasibility to be used in organ transplantation without risk of rejection and need of immunosuppression [14,31]. Few highlights on the therapeutic potential of iPSCs have been summarized in Table 2. The focus of the current review is to highlight and discuss the therapeutic roles of human iPSCs in different conditions and the future.

### 1.1. Induced Pluripotent Stem Cells—The Niche Favoring Unique Aspects

Pluripotency and self-renewal are unique characteristics of iPSC that make them ideal for disease modelling and regenerative medicine. Their ability to indefinitely differentiate into cells of all the three germ layers makes them an important source for treating injuries as well as diseases. The availability of generating patient-specific iPSC with high efficiency and safety through protocols involving biochemical and epigenetic aspects expands the therapeutic potential of this tool. This can be assessed from the fact that a clinical trial involving iPSC-derived dopaminergic neurons have been initiated for Parkinson’s disease after successful in vivo studies involving immunodeficient mice highlighted no risk of tumorigenicity [43]. Further, tissue resident macrophages, which are critical for immunity and derived from human-iPSCs, have been found to be immunologically different and better than the traditional monocyte-derived macrophages. Studies have shown human iPSC macrophages to restrict *Mycobacterium tuberculosis* growth in vitro by >75%, and were found to be capable of mounting antibacterial response when challenged with pathogens [44]. The greatest niche for iPSCs is the ability to generate the same from different donor categories including the diseased, and healthy making its application in the clinical setting at any stage a feasibility without the ethical issues around the ESCs.

The fundamental use of iPSC in regenerative medicine remains undisputed, but the tumorigenic potential of residual undifferentiated stem cells necessitates the need to devise strategies to remove the same from differentiated cells. Different study reports multiple treatment methodologies for eliminating undifferentiated iPSCs and one such recent publication identified undifferentiated hiPSCs to be sensitive to treatment involving medium supplemented with high concentration of L-alanine [45]. Another study assessed the efficacy of plasma-activated medium (PAM) in eliminating undifferentiated hiSPCs through inducing oxidative stress. This study found PAM to selectively eliminate undifferentiated hiPSCs cocultured with normal human dermal fibroblasts, which were the differentiated cells. Lower expression of oxidative-stress related genes in the undifferentiated hiPSCs were found to be the underlying cause for PAM-selective cell death [46]. A recent study report describes the use of salicylic diamines to remove residual undifferentiated cells from iPSC-derived cardiomyocytes. Salicylic diamines were found to exert their specific cytotoxic activity in the pluripotent stem cells by inhibiting the oxygen consumption rate. Teratoma formation was also found to be abolished in comparison to untreated cells [47].

### 1.2. Application of iPSC in Cardiac Disease

Non-communicable diseases, including cardiovascular conditions, have emerged to be one of the leading causes for mortality in developed as well as developing nations. The trigger for myriad heart conditions exists both in genetics and the environment, which makes studying disease etiology in animal models complicated and inefficient. Animal model studies indicate up to 90% failure in new drug clinical trials, highlighting the limitation around prediction of safety and efficacy among humans. The iPSCs-based disease models have been studied for cardiac channelopathies including hereditary long QT syndrome (LQTS), dilated cardiomyopathy (DCM), hypertrophic cardiomyopathy (HCM), and arrhythmogenic right ventricular cardiomyopathy (ARVC); the endothelial cell disease including familial pulmonary arterial hypertension (FPAH); the smooth muscle cell condition including Williams-Beuren syndrome (WBS), and Marfan syndrome (MFS) [8].

LQTS is an inherited fatal arrhythmia syndrome and around 17 genes have been associated with congenital LQTS, including the three main genes; KCNQ1 (LQT1), KCNH2 (LQT2), and SCN5A (LQT3), together which account for ~75% of clinically definite cases. The current therapeutic intervention includes β-blockers and a surgical procedure named left cardiac sympathetic denervation. Though genetic markers have been defined, the occurrence of variance of unknown significance (VUS) in 1 of 3 patients adds to the dilemma of inconclusive diagnosis. The need for better diagnostic platforms to assess outcome of genetic variants as well as different therapeutics led to the introduction of iPSCs. Many studies have worked to improve the differentiation efficiency, cellular maturation, and lineage specificity, develop new high-throughput assays for cellular phenotyping, and promote clinical implementation of patient-specific genetic models. A study by Wu J.C. et al. [48], utilized patient iPSC-derived cardiomyocytes (iPSC-CMs) and devised various strategies to reduce heterogeneity. These include derivation of chamber-specific cardiomyocytes, cultivation for extended period, 3-dimensional and mechanical conditioning, rapid electric stimulation, and hormonal stimulation; use of multicellular preparations to reduce intercellular variability; and development of high-throughput cellular phenotyping using optogenetic sensors including genetically coded voltage and calcium indicators. Further, this study also established the utility of iPSC-CMs to distinguish between pathogenic and benign variants to improve diagnosis and management of LQTS using CRISPR genome editing. This study, using iPSC-CMs, also identified factors causative for prolonged QT including upregulation of genes; *DLG2*, *KCNE4, PTRF*, and *HTR2C* and downregulation of *CAMKV* gene. Thus iPSC-based model platforms aid in developing a better understanding around intractable clinical problems associated with diseases like LQTS.

In case of DCM, characterized by ventricular chamber enlargement, and dilation as well as systolic dysfunction, human derived iPSCs have been used to investigate the excitation-contraction-coupling machinery, response to positive inotropic interventions, and study the proteome profile. This study utilized DCM patient specific-iPSC derived from skin fibroblasts and identified defects in assembly and maintenance of sarcomeric structure in the mutated iPSC-CM, as well as lower response to β-adrenergic stimulation with isoproterenol, and increased [Ca^2+^] out and angiotensin-II. This indicates mutated CM from DCM patients to express blunted inotropic response [49]. In case of HCM which is the most common cause of sudden death among the young, iPSC models have been used to identify pathogenesis of the condition. Once such study involving iPSC-CM derived from patients in a maternally inherited HCM family positive for the mitochondrial 16s rRNA gene (MT-RNR2) mutation m.2336T > C identified mitochondrial dysfunction, and ultrastructure defects among the carriers. Further, reduction in levels of mitochondrial proteins, the ATP/ADP ratio, and mitochondrial potential was also found. These lead to increase in intracellular Ca^2+^ levels, that becomes causative for HCM-specific electrophysiological abnormalities [50]. Recent studies have also generated peripheral blood mononuclear cells-derived iPSC from HCM patient positive for the myosin binding protein C (MYBPC3) pathogenic mutation c.3369–3370 insC by the episomal method, which underwent successful differentiation to triblast cells with normal male karyotype, and expression of pluripotent markers indicating its usefulness as a tool to study HCM [51].

The iPSC models around FPAH have identified modification of BMPR2 signaling causing reduced endothelial cell adhesion, migration, survival, as well as angiogenesis. The autosomal dominant BMPR2 disease causing mutation has been found to be only 20% penetrant and the use of iPSC identified increased BIRC3 to be related to improved survival, indicating the potential to use protective modifiers of FPAH for developing treatment strategies in the future [52]. The iPSC model around WBS with haploinsufficiency found deficiency of elastin and the patient-derived smooth muscle cell to be immature and highly proliferative with defects in function and contractile properties. The rescue was done by upregulating elastin signaling and use of anti-proliferative drug rapamycin [53]. In case of MFS, disease pathogenesis investigation using iPSCs identified defects in fibrillin-1 accumulation, degradation of extracellular matrix, abnormal activation of transforming growth factor-β, and cellular apoptosis [54].

The iPSC technology is also largely viewed to promote pre-clinical drug trials and screening over animal models to overcome differences in electrophysiological properties between human and animal cardiomyocytes. Studies have shown patient-derived iPSCs to exhibit higher sensitivity towards cardiotoxic drugs that could be the cause for change in action potential and arrhythmia [55]. Studies which have analyzed the beat characteristics of 3D engineered cardiac tissues have proven the occurrence of physiologically relevant changes in cardiac contraction in response to increasing concentrations of drugs like verapamil (multi-ion channel blocker) and metoprolol (β-adrenergic antagonist) [56].

Thus, iPSC has been successfully used to model and understand pathogenesis of different cardiac diseases, providing insights on pathways around progression as well as for assessment of drug toxicity. These highlight the potential to use iPSC-based models for precision medicine in clinical use.

### 1.3. Application of iPSC in Degenerative Diseases

Theoretically iPSC has the potential to be programmed to form any cell in the human body, and coupled with improvements in reprogramming techniques, this technology has advanced our knowledge on disease pathology, developing precise therapeutics, as well as fuel advances in regenerative medicine [57]. In case of neurodegenerative conditions, and psychiatric disorders, the genetic predisposition and its relation to the disease pathophysiology is complex, and often there is alteration at structural as well as functional levels. In case of schizophrenia, which is aptly termed the “disease of the synapses”, studies have generated iPSC from family members positive for a frameshift mutation in schizophrenia 1 (*DISC1*) and used gene editing to generate isogenic iPS cell lines. This study found depletion of DISC1 protein among the mutation carriers, along with dysregulation of genes associated with synapses and psychiatric disorders in the forebrain. This mutation causes deficit of synaptic vesicles among the iPS-cell derived forebrain neurons. This identification of transcriptional dysregulation in human neurons, highlights a new facet involving synaptic dysregulation in mental disorders [58]. The technology of stem cell therapy has also been used to restore the functionality in many degenerative conditions including that of the retina that leads to loss of vision. Studies have evaluated the use iPSC to overcome challenges posed by use of stem cell therapy. The proposed strategy revolves around transplantation of photoreceptors with or without the retinal pigment epithelium cells for treating retinal degradation, with minimal risk using iPSC [59].

Degenerative disease generally progresses through multiple differentiation stages, and using iPSC models, these pathways of transition can be easily identified to assess cause as well as etiopathology better. Amyotrophic lateral sclerosis (ALS) involves loss of neurons from the spinal cord and motor cortex causing paralysis and death. The research around advancement of therapeutics, requires supply of human motor neurons positive for the causative genetic mutations that will also aid in understanding the root cause of motor neuron death. One study documented the production of iPS from ALS patient specific-skin fibroblasts from two sisters. Both were identified to be positive for the L144F (Leu144 → Phe) mutation of the superoxide dismutase (*SOD1*) gene that is associated with a slowly progressing form of ALS. This study found successful reprogramming to be possible with only four factors; *KLF4*, *SOX2*, *OCT4*, and *c-MYC*. Further, the severe disability state of the patients used for harvesting in this case did not seem to block the transformation process or efficiency [60]. Fanconi anemia (FA) is an inherited bone marrow failure syndrome and is a chromosomal instability disorder needing transplantation of hematopoietic grafts from HLA-identical sibling donors. The reduced quality of the hematopoietic stem cells from the bone marrow of the affected limits the benefit of gene therapy trials. Studies have worked upon formation of genetically corrected FA-specific iPSCs through non-hematopoietic somatic cells reprogramming to generate large number of genetically-stable autologous hematopoietic stem cells for treating bone marrow failure in FA. The reprogramming was done on dermal fibroblasts involving two rounds of infection with mouse-stem-cell-virus-based retrovirus encoding amino-terminal flag-tagged version of the four transcription factors; *OCT4*, *SOX2*, *KLF4*, *c-MYC*. A batch of genetically corrected somatic cells using lentiviral vectors encoding FANCA or FANCD2 was also used for reprogramming to overcome the predisposition to apoptosis found in FA cells. The FANCA involved fibroblasts also underwent successful transformation to generate iPSCs. This study also found restoration of the FA pathway as a necessity to generate iPS from somatic cells of FA patients. The persistent FANCA expression in the FA-iPS cells indicated successful generation of genetically corrected FA-iPSCs with functional FA pathway, and disease-free status [61].

Parkinson’s disease (PD) is a common chronic progressive disorder due to loss of nigrostriatal dopaminergic neurons. The pathophysiology of the disease is complex and research till date lacks complete understanding. Further, sporadic cases are not linked to any genetic variation. Development of patient-specific invitro iPSC models have been attempted to understand disease etiology better. Studies have worked upon generating iPSCs from sporadic cases of PD, which have been successfully reprogrammed to form dopaminergic neurons free of the reprogramming factors. This study utilized doxycycline-inducible lentiviral vectors that were excised with Cre-/lox-recombinase, resulting in generation of iPSC free of programming factors, and which retained all the pluripotent characteristics after removal of transgenes. This removal of promoter and transgene sequences from the vector reduced risk of oncogenic transformation and re-expression of the transduced transcription factors. This study highlighted the possibility of generating stable iPS-cell line in PD for better disease modelling [62]. Another study worked on improving the safety of human and non-human primate iPSC derived dopaminergic neurons for cell transplantation treatment in PD. This study found the protocol of NCAM(^+^)/CD^29^(low) sorting to result in enriching ventral midbrain dopaminergic neurons from the pluripotent stem cell-derived neural cell populations. Further, these neurons also exhibited increased expression of FOXA2, LMX1A, TH, GIRK2, PITX3, EN1, and NURR1 mRNA. These neurons were also found to bear the potential to restore motor function among the 6-hydroxydopamine lesioned rats, 16 weeks after transplantation. Further, the primate iPSC-derived neural cell was found to have survived without any immunosuppression after one year of autologous transplant, highlighting the proof-of-concept around feasibility and safety of iPSC-derived transplantation for PD [10].

Type 1 diabetes is an autoimmune condition involving destruction of the β-cells of the pancreas wherein transplantation with β-cells as islet tissues or the entire pancreas is suggested as an alternative over the traditional exogenous insulin supplementation. However, these come with risk of rejection, need of immunosuppression, apart from difficulty in the physiological control on blood glucose levels. To circumvent this block, generation of β-cells or islet tissues from human pluripotent stem cells like iPSCs has been attempted. Many studies have generated pancreatic β-like cells which secrete insulin in response to stimuli like potassium chloride [63]. However, co-excretion of glucagon, and somatostatin, apart from releasing unsuitable amounts of insulin; make these clinically inferior. iPSC-derived pancreatic endoderm cells have been shown to retain the potential to differentiate and are functionally comparable with adult β-cells. Further, the shortage of donor islet has been overcome using iPSCs, as pancreatic cells generated from these have been evaluated in clinical trials as a new source for transplantation therapy. The differentiation of iPSCs through mimicking the natural in vivo process was facilitated using a combination of growth factors including Nodal-activin, Wnt, retinoic acid, hedgehog, epidermal and fibroblast growth factor, bone morphogenetic protein, and Notch to activate as well as inhibit the key signaling pathway. This study thus highlighted the possibility of generating patient-specific fully functional pancreatic tissue for transplantation over donor islet for diabetes treatment [64].

These studies highlight the development around iPSCs and transplantation technology for treatment of degenerative diseases as well as use them as disease models. The ability to generate patient-specific iPSC from skin biopsies, increases safety of autologous transplants without risk of immunorejection.

### 1.4. Application of iPSC in Blood Disorders

The treatment for blood disorders involves need for mature red blood cells/erythrocytes from the bone marrow or umbilical cord blood, for blood transfusion, and is limited due to incompatibility in blood group and Rh antigens, and risk of infections [65]. Erythropoiesis is a complex process for generation of mature erythrocytes from the precursor erythroblasts that are difficult to culture in vitro, as the entire process occurs in the bone marrow mediated by complex interaction between cellular and extracellular environment involving hormones, cytokines, and growth factors [66]. Further, the fully differentiated red blood cells (RBCs) are not proliferative, and setting up a system for erythropoiesis-like maturation in precursor cells is a challenge. Further, recruitment of donors, need for rare blood group types, as well as safety in sensitive population groups, add to the roadblock [67]. Studies have investigated human pluripotent stem cells, including iPSCs as an alternative source for unlimited supply of functional erythrocytes. Studies have discussed different methods devised for RBC production, including using PSCs by repeating the developmental haematopoiesis; reprogramming somatic cells through transcription factors including OCT4, SOX2, c-MYC, KLF4, NANOG, LIN28; and stimulating the maturation of hematopoietic stem cells isolated from peripheral or umbilical cord blood [67,68]. The advantage of using iPSCs is their ability to differentiate into any cell type, and can be maintained indefinitely, thus becoming a potential source for cell replacement therapies. The potential of iPSc becomes highlighted by the fact that the French National Registry of People with a Rare Blood Phenotype/Genotype claims a single iPSc clone from their database could meet 73% of the needs of sickle cell disease patients [69]. This highlights that a limited number or RBC clones have the potential to supply to the majority needs of alloimmunized patients with rare blood groups.

Studies have also worked on developing iPSC models for blood malignancies including myelodysplastic syndromes (MDS), acute myeloid leukemia (AML), and myeloproliferative neoplasms (MPN). A study worked on generating iPSC clones from bone marrow and blood of patients by integrating mutational analysis with cell programming to generate different iPSC clones which represent different disease stage as well as spectrum of the diseases including predisposition, low- and high-risk conditions. Additionally, the researchers also utilized the CRISPR/Cas9 system to introduce as well as correct mutations in the iPSCs. This study found iPSC from AML patients upon differentiation exhibited the leukemic phenotype, and the derived hematopoietic stem cells contained two immunophenotypically distinct cell populations; an adherent and non-adherent fraction, wherein the adherent fraction cells continuously renewed and generated the non-adherent cells. The AML-iPSC thus generated was found to exhibit characteristics of the leukemia stem cell model thus becoming an efficient model for molecular analysis and studying key functional aspects to be utilized for developing better therapeutics [70]. In case of chronic myeloid leukemia (CML), the *BCR-ABL* gene fusion is the major disease driver, and treatment involves use of tyrosine kinase inhibitor (TKI), causing remission in the vast majority of the cases. Studies have shown the CML-iPSCs to not be affected by TKI even in presence of *BCR-ABL* expression, indicating absence of dependency in this state of differentiation. The CML-iPSCs factors essential for maintenance of *BCR-ABL* positive and iPSCs including phosphorylation of AKT, JNK, ERK1/2 remained unchanged while the expression of STAT5 and CRKL was decreased. Further, the hematopoietic cells derived from CML-iPSC regained TKI sensitivity thus facilitating understanding on the disease pathogenesis better [71,72]. In case of MDS, reprogramming to generate iPSCs has been done from patients with del7q mutation, which is the signature for the disease. The iPSCs with the mutation upon hematopoietic differentiation were found to generate low quantities of CD34+/CD45+ myeloid progenitor cells. Further, studying genetically engineered clones as well as the MDS-iPSC-del7q clone from the patient, the researchers functionally mapped MDS phenotype to regions 7q32.3–7q36.1, which is linked to loss of hematopoietic differentiation potential [73]. To highlight the efficiency of iPSC-technology in precision oncology, studies have also created isogenic iPSCs with del7q and mutation *SRSF2* P95L, each of these connected to a specific phenotype and drug response [74].

Human iPSC preclinical models also exist for monogenic blood disorders including thalassemia, and hemoglobinopathies for gene and cell therapy. Pilot trial investigations have explored the safety and effectiveness of mobilizing CD34^+^ hematopoietic progenitor cells in beta-thalassemia major adults. Further, the CD34^+^ were transduced with globin lentiviral vector, wherein the vector-encoded beta-chain was found to be expressed at normal hemizygous protein output levels in NSG mice. This trial thus validated an effective protocol for beta-globin gene transfer among thalassemia major CD34^+^ hematopoietic progenitor cells [75]. The risk of insertional mutagenesis using hematopoietic stem cells can be overcome through iPSCs which can be cloned and the clones with vector integration in the “safe harbor” sites become possible. The genomic safe harbors (GSHs) ensure that the inserted new genetic material functions as predicted, and do not cause any alterations to the host genome [76]. Studies have shown the use of gene editing tools in case of beta-thalassemia to not be successful in expression of beta-globin in the corrected locus, because of the developmental immaturity of the iPSCs. In such cases, insertion of globin gene copy in the GSH site like AAVS1 has been recommended as an alternative approach [77]. Human iPSC models for gene therapy have also been developed and studied for primary immunodeficiency syndromes, including chronic granulomatous disease (CGD) caused by mutations in genes which code for the phagocyte NADPH oxidase that produces reactive oxygen species (ROS) that kill bacteria. Studies have shown genetically corrected CGD-iPSCs from macrophages and neutrophils using CRISPR/Cas9 system in the single intronic mutation of the *CYBB* gene to exhibit antimicrobial activity through generation of ROS and phagocytosis [78].

Thus, the potential of iPSCs to study etiology of complex diseases which manifest late in life, as well as to identify markers for precision therapeutics, is worth exploring in the arena of clinical biomedical research. Human iPSC-based models are a true success in our understanding of disease pathogenesis away from the animal models.

### 1.5. Application of iPSC in Organ Dysfunctions

**Figure 3:**
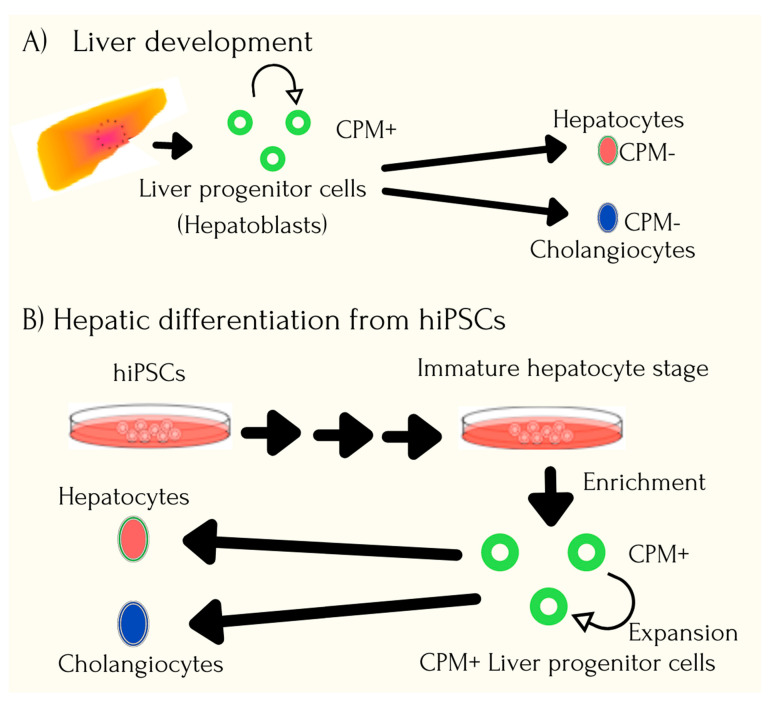
Process of liver development and hepatic differentiation from hiPSCs. The process of isolated cells from patients can be cultured and reprogrammed into patient-specific hiPSCs and quick comparison from natural liver development.

Organ donations are a key clinical need to treat end-stage organ failure conditions, and in often cases, patients are left to fight the acute shortage for the same. This apart, from identifying HLA-matched donors, handling risk of infections and rejection, as well as life-long immunosuppression, to a great extent damages quality of life for the affected as well as leads to loss of crucial time. Human iPSCs are being evaluated as a potential source for generating organs that can overcome roadblocks of shortage as well as risk of rejection. Studies have explored the possibility of generating a three-dimensional vascularized and functional liver organ from human iPSCs [79,80,81]. Generation of hepatocyte-like cells using iPSC technology has been reviewed to be fundamentally beneficial for treatment of severe liver disease, screening for drug toxicities, in liver transplantation, as well as to facilitate basic research [21]. Liver organogenesis involves delamination of specific hepatic cells from the foregut endodermal sheet to form a liver bud, which is then vascularized. One study prepared hepatic endoderm cells from human iPSCs through direct differentiation, wherein 80% of the treated cells were found to be positive for the cell fate determining hepatic marker; HNF4A. Further, to stimulate early organogenesis, the iPSCs were cocultured with stromal cells, human umbilical vein endothelial cells, and human mesenchymal stem cells, and after 48h of seeding, the human iPSCs were found to be self-organized into three-dimensional cell clusters visible macroscopically. This iPSC-derived liver bud, when further assessed by quantitative polymerase chain reaction (PCR) and microarray assay for expression analysis, highlighted the pattern to be similar to human fetal liver cell-derived liver buds. Hemodynamic stimulation to form organ was done by cranial window model, and the iPSC-derived tissue was found to perform liver-specific functions including protein synthesis and human-drug specific metabolism actions. This proof-of-concept study highlights the potential to use organ-bud transplantation for organ regeneration [82]. Figure 3 highlights the process of liver development and hepatic differentiation from hiPSCs.

Hepatocytes represent 80% of the liver mass and are the specialized epithelial cells crucial for maintaining homeostasis. The hepatic differentiation involves induction of endoderm differentiation by activin A, fibroblast growth factor 2 (FGF2), and bone morphogenetic protein 4 (BMP4), and such generated hepatocytes have been found to retain features of human liver including lipid and glycogen storage, urea synthesis, etc. Cholangiocytes in the inner space of the bile duct tree have also been generated from the common progenitor hepatoblast, through downregulation of signaling factors including epidermal growth factor (EGF), interleukin 6 (IL-6), Jagged 1, sodium taurocholate, and the generated cholangiocytes have been detected to express mature markers including SOX9 (SRY-Box Transcription Factor 9), OPN (Osteopontin), CK7 (Cytokeratin 7), CK19 (Cytokeratin 19), etc. The kupffer cells are the largest population of resident macrophages in the human body and also facilitate liver regeneration after an ischemic injury. Studies have demonstrated generation of iPSC-derived kupffer cells from macrophage precursors by adding a hepatic stimulus [83,84].

Another study evaluated lung regeneration by endogenous and exogenous stem cell mediated therapeutic approaches. Physiologically the tissue turnover rate in lung is slow and any insult to the regeneration process can lead to development of chronic obstructive pulmonary disease (COPD) as well as idiopathic pulmonary fibrosis. Bone marrow stem cells, embryonic stem cells, as well as iPSCs have shown excellent regenerative capacity to repair injured lung by generating whole lung in the lab using de-cellularized tissue scaffold and stem cells [85]. Lung organogenesis involves proximodistal patterning, branching morphogenesis, alveolarization, and cellular differentiation [86]. A study by Mou et al. [87], described generation of multipotent lung and airway progenitors from mouse ESCs and patient-specific cystic fibrosis (CF) iPSCs. The definitive endoderm from mouse ESCs were converted to foregut endoderm and then into replicating lung endoderm+Nkx2.1 (earliest marker of lung endoderm), which further transformed to a multipotent embryonic lung progenitor and airway progenitor cells. This study further highlighted that precise timing of the BMP, WNT, FGF signaling pathways are crucial for induction of NKX2.1. This study also utilized the same strategy to develop disease-specific lung progenitor cells from CF-iPSCs to make a model platform to study lung diseases. Further, the disease-specific lung progenitors were also engrafted in immunodeficient mice. One study derived lung progenitor cells with ~80% efficiency from iPSCs which differentiated onto alveolar epithelium both in vitro and in vivo. This study used Activin/BMP-4/bFGF treatment to obtain definitive endoderm from iPSC, which was further exposed to a series of pathway inhibitors (BMP, TGF-β, WNT), followed by longer exposure to FGF-19, KGF, BMP-4 and a small molecule CHIR99021 to mimic Wnt pathway to generate anterior foregut endoderm. The generated lung progenitors were further differentiated to many pulmonary progenitor cells including basal cells, goblet cells, ciliated cells, in vitro as well as in immunodeficient mice [88].

Studies have also utilized iPSC-derived organ models to study pathogenesis of the coronavirus disease-2019 (COVID-19). One study established a screening strategy to identify drugs that reduce angiotensin converting enzyme 2 (ACE2) using human ESCs-derived cardiac cells and lung organoids, as the infection occurs due to binding of the virus to ACE2 on the cell membrane. Target analysis revealed treatment with antiandrogenic drugs to reduce ACE2 expression, thus protecting the lung organoids from the SARS-CoV-2 infection. Clinical studies on COVID-19 identified patients with prostate disease, with elevated levels of circulating androgen to pose increased risk for high disease severity [89]. Another study utilized human lung stem-cell based alveolospheres to generate insights on SARS-CoV-2 mediated interferon response and pneumocyte dysfunction. This study described a chemically defined modular alveolosphere culture system for propagation and differentiation of the human alveolar type 2 (AT2) derived from primary lung tissue. The cultured cells were found to express ACE2 and transcriptome analysis of the infected alveolospheres were found to mirror features of the COVID-19 infected human lung, together with the interferon-mediated inflammatory response, loss of surfactant proteins, and apoptosis. Further, infected alveolospheres when treated with low dose interferons, a reduction in viral replication was noted. Thus, human stem-cell based models have also added insight to COVID-19 pathogenesis [90]. In case of use of iPSC three-dimensional model, a study by Huang et al. [91] found the derived AT2 to be susceptible to SARS-CoV-2 with decreased expression of surfactant proteins, and cell death, exhibiting delayed type I interferon response with multiplicities of infection of 5 and interferon-stimulated genes. Another study assessed inhibitor of SARS-CoV-2 infection using lung and colonic organoids from the gut. The derived iPSCs in three-dimensional, were positive for SARS-CoV-2 infection. In case of immune response, the tumor necrosis factor (TNF) and interleukin-17 (IL-17) signatures were noted after 24 h with multiplicities of infection of 0.1. This study also screened US Food and Drug Administration (USFDA) approved entry inhibitors including imatinib, mycophenolic acid, and quinacrine dihydrochloride; wherein treatment at physiologically relevant levels highlighted inhibition of SARS-CoV-2 infection both in iPSC-lung organoids and colonoids, indicating that iPSC models also prove to be a valuable source for safe drug screening [92].

Development of organ-specific progenitor cells which progress into the complete three-dimensional organ in a lab highlights the potential of iPSCs in regenerative medicine. Further, the impact of organ-system models to study infection pathology, highlights the wide clinical arena in which iPSC-technology can be used.

### 1.6. Application of iPSCs in Cancer Syndromes

The iPSCs have been generated for modelling pathogenesis of many diseases, and one of the most notable additions to the same is cancer, including models for familial cancer syndromes. One such study reports on the successful establishment of Li-Fraumeni Syndrome (LFS) patient-derived iPSC to study role of *p53* in development of osteosarcoma. LFS being a heterogenous cancer condition, osteosarcoma is one of the types wherein relevance of germline *p53* mutations have been highly reported. The pre-existing murine LFS models have been insufficient in charting the entire tumor landscape and patient-derived iPSCs in this regard have demonstrated the feasibility to effectively study human cancer syndromes. Studies have found the LFS-derived mesenchymal stem cells to exhibit low expression of targets of *p53* including p21 and MDM2; highlighting their ability to retain the defective *p53* function from the parental fibroblasts. Further, *p53* knockdown was found to cause upregulation of osteogenic markers in LFS osteoblasts, and the possibility to attain osteosarcoma-related phenotypes in LFS iPSC-derived osteoblasts was found. Further, gene expression analysis in LFS-derived osteoblasts was found to correlate with poor patient survival, and decreased time for recurrence. The impaired H19 restoration was also found to repress tumorigenic potential [36]. Another study involving modelling of osteosarcoma from LFS derived-iPSC identified the LFS osteoblasts to recapitulate oncogenic properties of osteosarcoma proving to be an excellent model to study disease pathogenesis [93]. In case of Noonan syndrome (NS) characterized by germline *PTPN11* mutations, studies which have derived hiPSCs from hematopoietic cells and which harbor the *PTPN11* mutations were found to successfully recapitulate features of NS. The iPSC-derived NS myeloid cells were found to exhibit increased STAT5 signaling and enhanced expression of micro-RNAs *viz*. miR-223 and miR-15a. Further, reducing miR-223 function was found to normalize myelogenesis, highlighting the role of micro-RNA dysregulation in early oncogenesis [94]. Human iPSC-derived hereditary cancer models have also aided in identifying *BRCA1*-deleted tumor niche to be the cause for disease progression [95].

The iPSC models around cancer aid in overcoming the hurdles posed by traditional cancer cell line systems, which may lose the characteristics of the original tumor with time, and further harnessing primary cancer cells at different stages of carcinogenesis is not feasible. The established iPSC reprogramming strategies can aid in differentiation of cancer cells to target cell lineages which can aid in studying each of the different stages in cancer progression [96]. The iPSCs developed from primary tumors, as well as cancer cell lines are invaluable tools to study genetic alterations early-on in familial cancer syndromes which is crucial in understand disease pathogenesis. Apart from cancer cell lines, patient-derived xenograft models have also been proven to be efficacious for understanding tumor heterogeneity, genetic alterations, and testing efficacy of cytotoxic drugs. However, the need for successful engraftment, technical challenges, and variable growth rates, are the key limitations. Even in case of animal models, high rate of mortality, and absence of metastasis are the limitations [97,98,99]. Advancements in iPSC models have also led researchers to be able to design autologous iPSC-based vaccine which presents a broad spectrum of tumor antigens to the immune system of the mice, and also found success in eliciting a prophylactic reaction against multiple cancer types. These studies highlight the great promise iPSC-based autologous vaccines present towards cancer prevention as well as therapy [100].

## 2. Induced Pluripotent Stem Cells: Advantages and Beyond

The iPSCs plug the very fundamental requirement for advancing scientific and clinical research by aiding insight into the fundamental growth and regenerative processes that happen early-on in life. They have emerged as an efficient alternative to ESC in clinical settings especially for screening new pharmaceutical compounds. The ability to produce patient-specific iPSCs has made studying disease pathogenesis and experiments around treating and managing inherited disease conditions, a close reality. They provide an unlimited repertoire of highly differentiated cells from focus area, fueling multiple investigations without roadblock on availability. The fact that iPSCs have also demonstrated success in treating few conditions including substantial correction of SCA and FA, highlights their therapeutic potential [101]. With organ engineering, using patient-specific iPSCs, the possibility to produce animal-free meat are also few areas which are being rapidly explored. The areas where iPSCs have proven impact on investigations as well as therapeutics highlights the potential of the instrument in various fields of biomedicine. Research around safe application and minimizing involvement of animal cells is crucial to enhance usability factor among humans. The ability to generate and use iPSCs derived from large animals (e.g., non-human primates, swine, horses, sheep, and canines), being highly similar and homogenous to the human microenvironment, is currently made available along with simultaneous generation of resources for improvement of animal welfare.

Scientific and technological advancements have also aided in designing high throughput drug screening systems based on hiPSC stem cell-derived atrial myocytes for detecting cardiac toxicity in atrial arrhythmias. The platform established by human iPS-derived cardiomyocytes was found to be useful in detecting propensity for drug-induced tachyarrhythmias [102]. The potential of iPSCs have also empowered COVID-19 studies wherein iPSC-derived airway epithelial cells were successfully developed to models consisting of cell types found in human upper airway epithelium. Further, the cells were found to secrete mucus and also exhibited positive for infection with COVID-19. The infected model also exhibited cytokine production at levels similar to a human infected body. This study highlights the significance of upper respiratory airway models in studying respiratory viral infections [103].

Although the impressive prospects of iPSC in clinical settings have been proven, the challenges that need to be realized and worked upon include tumorigenicity, heterogeneity, and immunogenicity. Teratoma formation continues to be serious challenge and a limiting factor in hiPSC transplantation. Though there are multiple elimination methods including cell sorting systems, antibody conjugates, the risk of tumor arising from proliferating progeny still exists. These apart, the reprogramming factors, and occurrence of genetic alterations during in vitro culturing also add to risk of tumorigenicity. Immunogenicity characterized by rejection especially in allograft transplantation deems the need to use immunosuppressants. Heterogeneity has also been reported as a limitation in iPSC which to a great extent is an outcome of genetics and differences in gene expression [104]. Apart from these, studies have also noted variability and reproducibility of results across multiple laboratories as a drawback. Different reprogramming methods, chromosomal instability, can impact the phenotype of the generated iPSC. Further, many protocols described and published today to a great extent result in formation of immature iPSCs and the existing advancements for inducing phenotype-specific maturation has been recorded to involve few months. Further, the need for broad range of iPSCs with respect to disease as well as cohort used for iPSC generation is a need [105].

## 3. ESCs and iPSCs in Clinical Trials

Induced pluripotent stem cells are also now being investigated in clinical trials for diseases and one such involves the Hirschsprung disease initiated in the year 2020. This case-control involves use of patient-derived iPSCs to develop models of the disease to understand genetic factors contributing to disease pathogenesis [106]. The global distribution of clinical studies indicates United States of America (USA) to be leading in the numbers at 36%, followed by France and China at 15%, and 12% respectively. Further, in case of interventional studies involving re-transplantation of the generated iPSCs into humans, China was found to be leading at 36.7%, while records find USA to have 16.7%. In case of target disease conditions being investigated, ophthalmic diseases ranked highest at 24.4%, followed by non-communicable ailments at 22.1%, cardiovascular diseases at 14.5%, and neurological diseases at 13% [107]. Few clinical trials involving ESCs/iPSCs have been listed in Table 3.

## 4. Conclusions

Human iPSCs have become a powerful tool in basic as well as translational and clinical research because of their ability to be maintained indefinitely whilst preserving the genetic makeup of the host. Further, technological advancements which enable genetic manipulation of iPSCs, ensure they become a rich repertoire for cell replacement therapy. Identification of GSH regions further adds to the feasibility to reduce risk of transplantation to a great extent. Their ability to provide cell-specific information, makes studying interaction between genetics, epigenetics, as well as extracellular environment a possibility. Complex relationships involving multiple cell types becomes dissectible with iPSC-models, and their ability to harbor large chromosomal alterations as well as deletions, makes them a favorable model for cancer studies. Though highly researched upon, the technological advancements to decipher the impact of genetic and epigenetic alterations as well as variability in cell clones or colonies is still naïve. Moreover, development of engraftable cell lines need to be devised considering the safety and efficacy aspect.

## Figures and Tables

**Table 1:** Reprogramming strategies for iPSCs in human species. Various programming strategies with ensuring safety and quality for therapeutic applications include the integrative or nonintegrative transfer systems using viral or nonviral.

Vector	Cell Type	Genes	Efficiency	Reference
Retrovirus	Skin fibroblasts	OCT4, SOX2, KLF4	0.001%	[18]
	Fibroblasts	OCT4, SOX2 and Valproic acid	0.001%	[19]
	Skin cancer cell line	*miR-302*	Unknown	[20]
Lentivirus	Embryonic fibroblasts	OCT4, SOX2, NANOG, LIN28	0.01%	[21]
	Fibroblasts	OCT4, SOX2, KLF4, c-MYC	0.01%	[22]
Adenovirus	Embryonic fibroblasts	OCT4, SOX2, KLF4, c-MYC	0.0002%	[23]
Sendai virus	Cord blood CD34+ cells	OCT4, SOX2, KLF4, c-MYC	0.2%	[24]
Recombinant protein	Fibroblasts	OCT4, SOX2, KLF4, c-MYC	0.001%	[25]
mRNA	Fibroblasts	OCT4, SOX2, NANOG, LIN28	0.05%	[26]

Note: OCT4, Octamer-binding Transcription Factor 4; *SOX2*, Sex-determining Region Y box 2; KLF4, Kruppel-like factor 4.

**Table 2:** Few highlights of iPSC-disease models and the investigated therapy. The example of therapeutic potential of iPSC towards personalized cell therapy and disease modelling, has extended the functionality of the pluripotency beyond laboratory tables as a research tool in murine and human models.

Disorder	iPSC Characteristic	Therapy	Reference
Chronic Granulomatous Disease (CGD)—Preclinical	CGD-iPS-cells which transformed to neutrophils lacked production of reactive oxygen species (ROS)	The zinc finger nuclease -mediated functional correction of the causative *CYBB* gene in the neutrophils restored ROS production.	[32]
Hemophilia A (HA)—Preclinical mice model	The HA-iPSC derived endothelial cells lacked *F8* gene expression and secretory protein	Lentiviral-based vector with *F8* transgene and driven by endothelial-specific promoter was used, and the derived endothelial cells exhibited restored *F8* gene expression	[33]
Hemophilia B (HB)—Preclinical hemophilic mice model	HB-iPSC derived hepatocyte-like cell lacked secretion of clotting factor IX	The CRISPR/Cas9 gene editing system was used to correct the cDNA in the HB-iPSCs and the resultant hepatocyte-like cells exhibited restored synthesis ability for clotting factor IX.	[34]
Severe combined immunodeficiency (SCID)—Preclinical	SCID-iPSCs with JAK3 deficiency exhibited lack of early T cell development	Gene editing by CRISPR/Cas9—enhanced gene targeting was used to correct the JAK3 mutation, which restored normal T cell development along with production of mature T cells with a broad T cell receptor repertoire.	[35]
Thalassemia—Preclinical	The iPSC-derived erythroid cells from homozygous alpha thalassemia exhibited lack of expression of the alpha globin gene	Zinc finger nuclease-mediated insertion of the globin transgene was done in the safe harbor site; AAVS1 on human chromosome 19 for correction of alpha-thalassemia major hydrops fetalis. The homozygous insertion corrected the imbalance of the globin chain in the erythroid cells.	[36]
Young-onset Parkinson’s disease (YOPD)—Preclinical	YOPD-iPSCs were differentiated to midbrain dopaminergic neural culture that exhibited increased accumulation of soluble α-synuclein protein and phosphorylated protein kinase Cα, and reduced abundance of the lysosomal membrane protein LAMP1	Activation of lysosomal-specific pathway by phorbol ester PEP005 reduced α-synuclein, and phosphorylated protein kinase Cα levels, and increasing LAMP1 levels.	[37]
Parkinson’s disease (PD)—Proof-of-concept rodent study	Human iPSC-derived midbrain dopaminergic neurons were subjected to sorting to enrich the ventral midbrain (VM) neurons and improve efficacy and safety of cell therapy	Sorting using NCAM(+)/CD29(low) enriched VM dopaminergic neurons better. Further, PiPSC-derived NCAM(+)/CD29(low) DA neurons were able to restore motor function of 6-hydroxydopamine (6-OHDA) lesioned rats 16 weeks after transplantation.	[10]
Alzheimer’s disease (AD)—Proof-of-concept preclinical study	AD patient-derived iPSCs were carriers of three copies of the amyloid precursor protein (*APP*) gene	Gene editing by CRISPR/Cas9 system enables generation of iPS-cell lines with monoallelic, biallelic, or triallelic knockout of APP. The corticol neurons generated from isogenically corrected iPSCs were found to exhibit gene-dosage correlation dependent disease-phenotype correlation.	[38]
Amyotrophic lateral sclerosis (ALS)—Preclinical	ALS-iPSCs from fibroblasts exhibited SOD1+/A272C and FUS+/G1566A mutations	The CRISPR/Cas-9 nickases was used to correct the mutation and the gene corrected ALS-iPSCs (FUS+/+ and SOD1+/+) exhibited all pluripotency markers including OCT4, NANOG, and SOX2.	[39]
Barth syndrome (BTHS)—Proof-of-concept preclinical study	The BTHS-iPSC-derived cardiomyocytes exhibited abnormalities associated with mutations in the *TAZ* gene. Further, the cardiomyocytes assembled sparsely, and exhibited irregular sarcomeres.	The CRISPR/Cas9 system was used to introduce *TAZ* gene mutation in healthy donor iPSC-derived cardiomyocytes to identify relationship. Further, administration of antioxidant mitoTEMPO in the BTHS-iPCs-derived cardiomyocytes exhibited suppression of excess ROS production and normalization of the sarcomere organization and contractility.	[40]
Long QT syndrome (LQTS)—Preclinical	The LQT15-iPSC with *CALM2-*N98S mutation were differentiated into cardiomyocytes exhibited significantly lower beating rates, prolonged AP durations, and impaired inactivation of LTCC currents	The CRISPR/Cas9 system was used to correct the mutation in *CALM2* and the resultant gene corrected iPSC-derived cardiomyocytes showed reversal in electrophysiological abnormalities with successfully recapitulating the disease phenotype.	[41]
Cystic fibrosis (CF)—Preclinical	The CF-iPSCs were positive for the *CFTR* mutation involving homozygous deletion of F508	The CRISPR/Cas9 system was used to correct the *CFTR* mutation, in combination with a completely excisable selection system. The gene correct iPSCs successfully differentiated to mature airway epithelial cells and recovered normal CFTR expression.	[42]

**Table 3:** Summary of few clinical trials involving ESCs/iPSCs from ClinicalTrials.gov (accessed on 17 May 2021).

ID Number	Disease	Cell Type	Title	Intervention	Country
NCT03763136 [108]	Heart disease	hiPSCs-derived cardiomyocytes	Epicardial Injection of Allogeneic Human Pluripotent Stem Cell-derived Cardiomyocytes to Treat Severe Chronic Heart Failure	Injection into the myocardium	China
NCT03119636 [109]	Parkinson’s disease	hESC-derived neural precursor cells	A Phase I/II, Open-Label Study to Assess the Safety and Efficacy of Striatum Transplantation of Human Embryonic Stem Cells-derived Neural Precursor Cells in Patients with Parkinson’s Disease	Stereotaxic intra-striatal injection	China
NCT03877471 [110]	Primary ovarian insufficiency	hESC-derived mesenchymal stem cells-like cells	Safety Study of Human Embryonic Stem Cell Derived Mesenchymal Stem Cell (MSC)-Like Cells Transplantation in Women with Primary Ovarian Insufficiency (POI)	Injection into ovaries	China
NCT03841110 [111]	Advanced solid tumors	iPSC-derived NK cell cancer immunotherapy	FT500 as monotherapy and in combination with immune checkpoint inhibitors in subjects with advanced solid tumors (Phase 1)		USA
NCT03403699 [112]	Diabetic Retinopathy	iPSC-derived mesoderm cells	Human iPSC for Repair of Vasodegenerative Vessels in Diabetic Retinopathy (iPSC)	injection into the vitreous cavity of diabetic rodents and primate eyes	USA
NCT03222453 [113]	Beta thalassemia	Hematopoetic stem cells from beta-thalassemia iPSCs	Thalassemia Treatment Based on the Stem Cell Technology		China
NCT00874783 [114]	Neurodegenerative Disorders	iPS cells from cell cultures from skin biopsies or the patient’s hair	Derivation of Induced Pluripotent Stem Cells from Somatic Cells Donated by Patients with Neurological Diseases for the Study of the Pathogenesis of the Disorders and Development of Novel Therapies		Israel
NCT02772367 [115]	Breast cancer	iPSC-derived cardiomyocytes	Generation of Induced Pluripotent Stem Cell Derived Cardiomyocytes from Patients Exposed to Trastuzumab Therapy for Breast Cancer		USA
NCT01691261 [116]	Acute Wet Age-Related Macular Degeneration	hESC-derived retinal pigment epithelium	Phase 1, open-label, safety and feasibility study of implantation of PF-05206388 (Human embryonic stem cell derived retinal pigment epithelium (RPE) living tissue equivalent) in subjects with acute wet age-related macular degeneration and recent rapid vision decline	Intraocular use of retinal pigment epithelium living tissue	United Kingdom (UK)
